# Efficacy of different preoperative antiseptics in preventing a risk of surgical site infections: a systematic review and meta‑analysis of randomized controlled trials

**DOI:** 10.20452/wiitm.2024.17885

**Published:** 2024-07-23

**Authors:** Lutao Yang, Shunxin Liao, Qing Cao, Sanjay Rastogi

**Affiliations:** Department of Burn Plastic and Wound Repair, Jiujiang City Key Laboratory of Cell Therapy, Jiujiang No.1 People’s Hospital, Jiujiang, China; Boston University Medical Campus, Boston, Massachusetts, United States

**Keywords:** surgical site infections, preoperative skin antiseptics, postoperative wound infection, chlorhexidine alcohol, various surgeries

## Abstract

**INTRODUCTION:**

Surgical site infection (SSI) is a predominant postoperative complication that markedly increases health care expenses. Published systematic reviews, meta‑analyses and international guidelines vary in their recommendations for the most effective preoperative skin antiseptic solutions and their concentrations.

**AIM:**

The aim of this study was to assess the efficacy of different preoperative antiseptics for prevent‑ ing the risk of SSIs.

**MATERIALS AND METHODS:**

A complete search was conducted using PubMed, EMBASE, Scopus, and Cochrane Library databases to collect peer‑reviewed articles.

**RESULTS:**

This meta‑analysis included 10 587 surgical patients from 18 randomized clinical trials to determine the effectiveness of chlorhexidine in alcohol (CHA; 0.5%, 2%–2.5%, and 4%) with aqueous or alcoholic iodine in preventing postoperative SSIs. This meta‑analysis found that 2%–2.5% CHA is the most effective preoperative antiseptic for preventing SSIs, with significant reduction in their incidence and significant antimicrobial activity.

**CONCLUSIONS:**

The findings of this meta‑analysis indicate that for patients undergoing any type of surgery, the use of 2%–2.5% CHA for skin preparation is the most effective method for preventing SSIs.

## INTRODUCTION

Surgery is a medical discipline that employs manual and instrumental techniques to diagnose or treat pathological condi‑ tions, alter bodily functions, reconstitute or improve esthetics and appearance, or eliminate undesirable tissues or foreign bodies.^1^ Minimally‑invasive surgery is currently a preferred method resulting in quicker recovery and smaller incisions. Despite the benefits of smaller blood loss, decreased discomfort, and minimized scarring, as compared with standard surgery, there is still a risk of surgical site infections (SSIs).^2^ SSI is the most common postsurgery complication, and is associated with increased morbidity, mortality, and hospital expenses.^3^ Approximately 14%–16% of all hospital‑acquired infections are SSIs, making it the second most common type of infections. Approximately 7%–9% of people encounter postsurgical complications due to an infection.^4^ The infection rate shows substantial variability depending on patient‑related factors, such as socioeconomic status, medical disorders, weakened immune systems, use of steroids, hemorrhage, body mass index, duration of operation, lack of preventive measures, and emergency surgical procedures.^5^ Multiple exogenous factors influence the occurrence of SSIs, including the patient’s skin preparation, hand hygiene practices, operating room conditions, instrument sterilization procedures, and the use of hospital supplies.^6^^;^^7^ Hence, choosing a suitable pre‑operative antiseptic for skin preparation is a crucial factor in preventing SSIs. Medical professionals generally acknowledge and consider preoperative skin antiseptics a routine practice due to their efficacy in preventing SSIs, but there is an ongoing discussion about the most effective preoperative antiseptic. The recommendations provided by the World Health Organization (WHO),^8^ the United Kingdom National Institute for Health and Care Excellence (NICE),^9^ and the United States Centers for Disease Control and Prevention (US CDC)^10^ offer conflicting guidance regarding appropriate surgical skin preparation methods. The NICE and WHO guidelines recommend chlorhexidine combined with alcohol (CHA), whereas the US CDC advises the use of any solution, including alcohol. Several randomized controlled trials (RCTs) have been conducted to provide recent evidence on this topic.^11^^;^^12^ These RCTs often evaluated various formulations and concentrations of skin antiseptics. Several meta‑analyses have been completed, including those carried out for the development of the NICE and WHO guidelines.^13^^;^^14^ Nevertheless, these evaluations demonstrated discrepancies in both the choice of studies and the data, and none of the guidelines offer a precise prescription regarding a suitable concentration of the suggested antiseptics. Previous studies showed that alcohol‑based preoperative skin antiseptics significantly reduce the occurrence of SSIs. Povidone‑iodine (PI) and CHA are well‑studied skin disinfectants thanks to their efficacy against a broad spectrum of pathogens, encompassing gram‑positive bacteria, gram‑negative bacteria, viruses, fungi, and Mycobacterium tuberculosis.^15^ Therefore, we conducted a meta‑analysis and systematically reviewed the findings of 18 RCTs,^16^^;^^17^^;^^18^^;^^19^^;^^20^^;^^21^^;^^22^^;^^23^^;^^24^^;^^25^^;^^26^^;^^27^^;^^28^^;^^29^^;^^30^^;^^31^^;^^32^^;^^33^ which compared different concentrations of CHA (0.5%, 2%–2.5%, and 4%) with aqueous or alcoholic iodine, selected according to pre‑established inclusion and exclusion criteria.

## AIM

The purpose of this systematic review and meta‑analysis was to evaluate the effectiveness of various preoperative antiseptics in reducing the likelihood of SSIs.

## MATERIALS AND METHODS

### Search strategy and selection criteria 

This systematic review and meta‑analysis adhered to the reporting guidelines outlined in the Preferred Reporting Items for Systematic Reviews and Meta‑Analysis statement.^34^ We conducted a systematic review of RCTs that compared the effectiveness of 2 antiseptic skin preparations, namely CHA and iodine in aqueous and alcohol‑based solutions. The study focused on the effects of different concentrations of CHA in preventing the SSIs in patients aged at least 18 years undergoing surgery. The primary outcome of interest was the reported incidence or rates of SSIs, antimicrobial effect of the antiseptics, and adverse events associated with their use. We excluded trials involving children, animal studies, studies that were not randomized, and those that did not provide normal preoperative intravenous antibiotic prophylaxis. There were no limitations regarding language or year of the publication. We searched the scientific literature databases of Embase, PubMed, Scopus, and Cochrane CENTRAL for publications published until March 30, 2024. The search terms used were: “postoperative surgical site infections” OR “surgical site infections” OR “SSI” OR “skin antiseptic” OR “preoperative skin antiseptics” OR “chlorhexidine‑alcohol” OR “CHA” OR “2%–2.5% CHA” OR “4% CHA” or “0.5% CHA” OR “aqueous iodine” OR “alcoholic iodine” OR “preoperative care” OR “postoperative wound infection” OR “antiseptic” OR “general surgery” OR “caesarean section” OR “clean surgery” OR “nonclean surgery” OR “incidence / rate of surgical site infection” OR “adverse events” OR “RCT” OR “randomized controlled trial” OR “wound classification” OR “systematic review” OR “meta‑analysis”. Following the PICO (Patient, Intervention, Comparison, Outcome) criteria,^35^ we identified and assessed keywords for agreement in both the Medline and EMBASE databases. The specified keywords were entered into the title / abstract/ keyword field during the Scopus search. The search terms “surgical site infections,” “preoperative skin antiseptics,” and “incidence of postoperative surgical site infections” were used in the Cochrane database.

The utilization of the PICO framework allowed us to create specific selection criteria. The letter “P” was used to designate patients who underwent surgery. The intervention group utilized different concentrations of CHA (0.5%, 2%–2.5%, or 4%) to prevent SSIs. The letter “C” was used for a control group treated with alcoholic or aqueous iodine. The primary clinical outcomes, denoted by “O”, consisted of the overall incidence of SSIs, antimicrobial effects of the antiseptic, and any adverse events associated with its use. Our study only included RCTs. Additional publications were identified by conducting backward and forward citation monitoring on previously published meta‑analyses and included research. The comprehensive search approach is defined in TABLE 1. Two reviewers independently assessed the titles, abstracts, and full texts of papers that potentially met the inclusion criteria. Any inconsistencies were resolved through a discussion and, if necessary, the senior author (MAB) was consulted.

**TABLE 1:** Database search strategy

Database	Search strategy
Scopus	“postoperative surgical site infections” OR “surgical site infections” OR “SSI” OR “skin antiseptic” OR “preoperative skin antiseptics” OR “chlorhexidine alcohol” OR “CHA” OR “2%–2.5% CHA” OR “4% CHA” or “0.5% CHA” OR “aqueous iodine” OR “alcoholic iodine” OR “preoperative care” “postoperative wound infection” OR “antiseptic” OR “general surgery” OR “caesarean section” OR “clean surgery” OR “nonclean surgery” OR “incidence / rate of surgical site infection” OR “adverse events” OR “RCT” OR “randomized controlled trial” OR “wound classification” OR “systematic review” OR “meta‑analysis” 1 AND 2
PubMed	“postoperative surgical site infections” OR “surgical site infections” OR “SSI” [MeSH Terms] OR “skin antiseptic” OR “preoperative skin antiseptics” [All Fields] OR “chlorhexidine alcohol” [MeSH terms] OR “CHA” [All fields] OR “2%–2.5% CHA” OR “4% CHA” or “0.5% CHA” OR “aqueous iodine” [All Fields] OR “alcoholic iodine” [All Fields] OR “preoperative care” [All fields] “postoperative wound infection” [MeSH Terms] OR “antiseptic” [All Fields] OR “general surgery” [All Fields] OR “caesarean section” [All Fields] OR “clean surgery” OR “nonclean surgery” OR “incidence / rate of surgical site infection” [All Fields] OR “adverse events” [All Fields] OR “RCT” [All Fields] OR “randomized controlled trial” [All Fields] OR “wound classification” OR “systematic review” [All Fields] OR “meta‑analysis” [All Fields] 1 AND 2
Embase	“postoperative surgical site infections” / exp$ OR “surgical site infections” / exp$ OR “SSI” / exp$ OR “skin antiseptic” / exp$ OR “preoperative skin antiseptics” / exp$ OR “chlorhexidine alcohol” / exp OR “CHA” / exp OR “2%–2.5% CHA” / exp OR “4% CHA” / exp or “0.5% CHA” / exp OR “aqueous iodine” / exp OR “alcoholic iodine” / exp OR “preoperative care” / exp “postoperative wound infection” / exp OR “antiseptic” / exp OR “general surgery” / exp OR “caesarean section” / exp OR “clean surgery” / exp OR “nonclean surgery” / exp OR “incidence / rate of surgical site infection” / exp OR “adverse events” / exp OR “RCT” / exp OR “randomized controlled trial” / exp OR wound classification” OR “systematic review” / exp OR “meta‑analysis” / exp 1 AND 2
Cochrane library	“postoperative surgical site infections): ti, ab, kw OR “surgical site infections”: ti, ab, kw OR “SSI”: ti, ab, kw OR “skin antiseptics”: ti, ab, kw OR “preoperative skin antiseptics”: ti, ab, kw OR “chlorhexidine alcohol” ti, ab, kw OR “CHA”: ti, ab, kw OR “2%–2.5% CHA”: ti, ab, kw OR “4% CHA”: ti, ab, kw OR “0.5% CHA”: ti, ab, kw OR “aqueous iodine”: ti, ab, kw OR “alcoholic iodine”: ti, ab, kw OR “preoperative care”: ti, ab, kw (word variations were searched) “postoperative wound infection”: ti, ab, kw OR “antiseptic”: ti, ab, kw OR “general surgery”: ti, ab, kw OR “caesarean section”: ti, ab, kw OR “clean surgery”: ti, ab, kw OR “nonclean surgery”: ti, ab, kw OR “incidence / rate of surgical site infection”: ti, ab, kw OR “adverse events”: ti, ab, kw OR “RCT”: ti, ab, kw OR “randomized controlled trials”: ti, ab, kw OR “systematic review”: ti, ab, kw OR “meta‑analysis”: ti, ab, kw OR (word variations have been searched) 1 AND 2

Abbreviations: ab, abstract; CHA, chlorhexidine alcohol; kw, keyword; MeSH terms, medical subject heading; $ exp: explosion in Emtree searching of selected subject terms and related subjects; ti, title

### Data analysis

This work includes studies that provided comparative data on the effectiveness of different concentrations of CHA and aqueous or alcoholic iodine solutions in preventing SSIs. The studies were chosen based on full texts meeting the inclusion criteria and having sufficient data for a 2 × 2 table. Outdated, anecdotal, or entirely expert‑based bibliographic references were excluded from the examination process. Two researchers independently collected the demographic profiles of the patients and event data, including relevant components, from the studies included in the analysis. The data were obtained using a predetermined form and included information on the author, year and country of publication, primary outcomes, secondary outcomes, number of patients in each study group, type of surgery, number and type of SSIs, adverse events, definition of SSI, and surgical wound classification. If the data provided by the authors were inadequate or unclear, they were contacted to obtain additional information. For example, if the concentration of the antiseptic solution was unknown, clarification was sought. The main result assessed was the overall incidence or occurrence of SSIs, antimicrobial activity of the antiseptics, and any adverse effects resulting from the intervention, such as allergic reactions, pain, skin irritation, or erythema.

### Risk of bias assessment

The researchers utilized a standardized questionnaire to assess the included studies for any possible bias. Two authors independently evaluated the risk of bias utilizing the Cochrane risk‑of‑bias tool, version 2.^36^ The tool consisted of 5 components: randomization‑induced bias, bias due to deviations from intended interventions, bias attributed to missing outcome information, bias during outcome evaluation, and bias in selecting the reported outcomes. An additional reviewer took on the responsibility of resolving any arising conflicts. Ultimately, a possible bias was assessed and categorized as “uncertain risk,” “high risk,” or “low risk.” Small‑study effects and publication bias were assessed using a comparison‑adjusted funnel plot.^37^ The significant effect of this bias was confirmed using the Egger regression test^38^ conducted with MDCalc software.^39^

### Statistical analysis

The software program Review Manager (RevMan) 5.4^40^ was employed to evaluate and analyze the influence of different continuous and dichotomous results. For each study, risk ratio (RR) and 95% CIs^41^ were computed to assess binary outcomes. The DerSimonian and Laird method^42^ was employed to calculate the RR using a 2 × 2 table^43^ consisting of event data. Studies that did not report any SSIs in either group were omitted from the quantitative evaluation. Forest plots^44^ were designed to evaluate the influence of different outcome determinants. Statistical methods, such as the I^2^ test^45^ and the χ^2^ test,^46^ accompanied with a *P* value, were used to assess heterogeneity. Since the investigations were conducted under different settings, a random effect model^47^ was used. The *P* value below 0.05 was deemed significant.^48^ A hierarchical summary receiver operating characteristic curve (HSROC) plot^49^ was generated to evaluate the test accuracy of all included studies. A subgroup analysis was performed to assess the effectiveness of various concentrations of CHA and aqueous / alcoholic iodine in terms of overall incidence of SSI, antimicrobial effects, and associated adverse events in different types of surgeries.

### Ethics

All procedures performed in the study were in accordance with the institutional and / or national research committee standards (IRB 20241095) and with the 1964 declaration of Helsinki and its later amendments or comparable ethical standards.

## RESULTS

### Study selection outcomes

**FIGURE 1:**
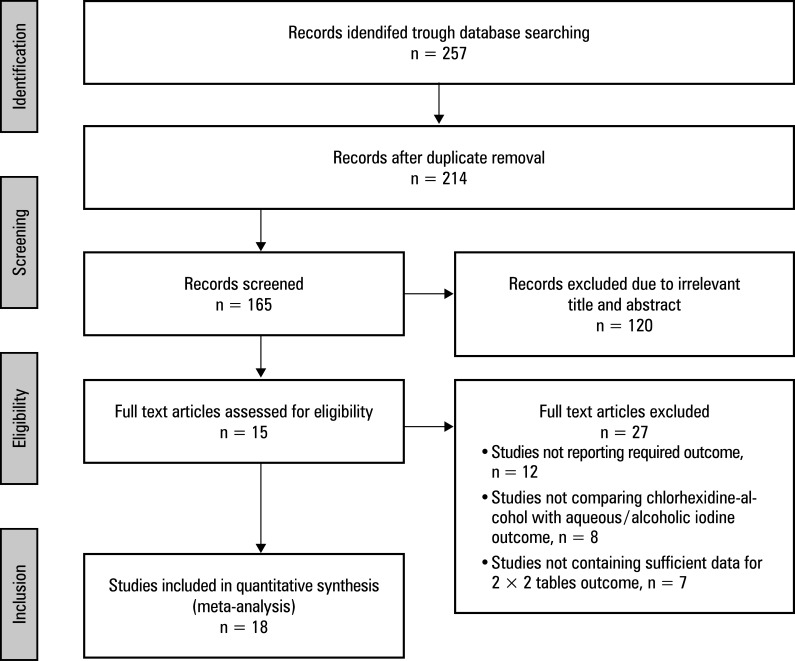
Preferred Reporting Items for Systematic Reviews and Meta-Analyses (PRISMA) study flow diagram

An exhaustive electronic survey was conducted across multiple databases, and 257 studies were identified as meeting the inclusion criteria outlined in the PICO paradigm. A total of 214 articles were selected for consideration, while 53 papers were excluded due to duplicate content and inapplicable titles and abstracts. Following further screening, 165 papers were assessed for eligibility. However, after applying the inclusion‑exclusion criteria, 120 studies were found ineligible and were excluded. The remaining 45 articles were then evaluated to ascertain their eligibility. Of those, 27 were excluded for failing to meet the inclusion criteria, lacking sufficient data to generate 2 × 2 tables, or lacking significant outcome measures. Finally, as shown in FIGURE 1, 18 RCTs that satisfied the predetermined inclusion‑exclusion criteria were included in this meta‑analysis. They investigated a total of 10 587 participants aged at least 18 years. Two studies^16^^;^^17^ compared the effectiveness of 4% CHA vs aqueous iodine, 7 studies^18^^;^^19^^;^^20^^;^^21^^;^^22^^;^^23^^;^^24^ of 2%–2.5% CHA vs 9 of aqueous iodine^16^^;^^18^^;^^19^^;^^20^^;^^21^^;^^24^^;^^25^^;^^28^^;^^29^ 5 studies^25^^;^^26^^;^^27^^;^^28^^;^^29^ of 2%–2.5% CHA vs alcoholic iodine, 2 studies^30^^;^^31^ of 0.5% CHA vs aqueous iodine, and the remaining 2 studies(32,33) of 0.5% CHA vs alcoholic iodine in preventing the incidence of SSI. Demographic characteristics of the patients included in this meta‑analysis are detailed in TABLE 2 . Furthermore, event data for the 2 × 2 table were retrieved from the studies to perform this meta‑analysis.

**TABLE 2:** Characteristics of the included randomized controlled trials (continued on the next page)

Author	Total participants	Age, y	Participants in CHG/IG groups	SSI/N total	Treatment 1	Treatment 2	Type of surgery	Wound classification	Primary and secondary outcomes	SSI definition^a^
4% Chlorhexidine alcohol vs aqueous iodine										
Gezer et al^16^	110	≥18	55/55	17/110	4% CHG with alcohol 10%	PI (1% AI)	Gynecological surgery	Clean, nonclean surgery	OAI SSI, AE, AME	CDC
Paocharoen et al^17^	500	≥18	250/250	13/500	4% CHG in 70% IPA	10% PI (1% AI)	General surgery	Clean, nonclean surgery	OAI SSI, AE, AME	#H
2%–2.5% Chlorhexidine‑alcohol vs aqueous iodine										
Bibi et al^18^	388	≥18	157/151	34/388	20% CHG in 70% IPA	10% PI (1% AI)	General surgery	Clean, nonclean surgery	OAI SSI, AE, AME	CDC
Danasekaran et al^19^	120	50.02 (12.02)	60/60	16/120	2% CHG in 70% IPA	5% PI (0.5% AI)	General surgery	Clean, nonclean surgery	OAI SSI, AE, AME	#C
Kunkle et al^20^	60	≥ 18	33/27	3/60	2% CHG in 70% IPA	10% PI (1% AI)	Caesarean section	Clean, nonclean surgery	OAI SSI, AE, AME	#E
Luwang et al^21^	311	28.17 (4.75)	153/158	21/311	2% CHG in 70% IPA	10% PI (1% AI)	Caesarean surgery	Clean, nonclean surgery	OAI SSI, AE, AME	#B
Broach et al^22^	802	≥18	392 /396	172/ 802	2% CHG in 70% IPA	0.7% AI in 74% IPA	Colorectal surgery	Non‑clean surgery	OAI SSI, AE, AME	CDC
Springel et al^23^	932	≥18	461/471	62/932	2% CHG in 70% IPA	0.75% AI scrub + 10% PI paint (1% AI)	Caesarean section	Clean, nonclean surgery	OAI SSI, AE, AME	CDC
Xu et al^24^	159	≥18	79/80	3/159	2% CHG in 70% IPA	10% PI (1% AI)	Orthopedic surgery	Clean surgery	OAI SSI, AE, AME	#D
2%–2.5% Chlorhexidine‑alcohol vs alcoholic iodine										
Ngai et al^25^	1404	≥18	463/474	60/ 1404	2% CHG in 70% IPA	0.83% AI in 72.5% IPA	Caesarean section	Nonclean surgery	OAI SSI, AE, AME	CDC
Ritter et al^26^	279	≥18	112/167	26/279	2% CHG in 70% IPA	1% PI (0.1% AI) in 70% IPA	Orthopedic surgery	Clean surgery	OAI SSI, AE, AME	#F
Sistla et al^27^	556	≥18	278/278	33/ 556	2.5% CHG with 70% ethanol	10% PI (1% AI)	Inguinal hernia repair	Clean surgery	OAI SSI, AE, AME	CDC
Savage et al^28^	100	≥18	50/50	0/100	2% CHG in 70% IPA	0.7% AI in 74% IPA	Neurosurgery	Clean surgery	OAI SSI, AE, AME	None
Tuuli et al^29^	1147	≥18	572/575	84/1147	2% CHG with 70% IPA	8.3% PI (0.83% AI) in 72.5% IPA	Caesarean section	Clean, nonclean surgery	OAI SSI, AE, AME	#G
0.5% Chlorhexidine‑alcohol vs aqueous iodine										
Abreu et al^30^	56	≥18	30/26	10/56	0.5% CHG in alcohol	0.5% PI (0.05% AI)	Urological surgery	Clean, nonclean surgery	OAI SSI, AE, AME	CDC
Srinivas et al^31^	3510	≥18	1760/1750	50/3510	0.5% CHG in 70% IPA	5% PI (0.5% AI)	Upper abdominal surgery	Clean, nonclean surgery	OAI SSI, AE, AME	CDC
0.5% Chlorhexidine‑alcohol vs alcoholic iodine										
Perek et al^32^	94	≥18	54/40	6/94	0.5% CHG in 70% ethanol	50% propyl alcohol	Cardiac surgery	Clean surgery	OAI SSI, AE, AME	CDC
Shadid et al^33^	59	≥18	30/29	0/59	0.5% CHG in 70% alcohol	1% iodine in 70% alcohol	Orthopedic surgery	Clean surgery	OAI SSI, AE, AME	CDC

Abbreviations: AE, adverse event; AME, antimicrobial effect; CDC, United States Centers for Disease Control and Prevention; CHG, chlorhexidine-alcohol group; I, iodine; IG: iodine group; IPA, isopropyl alcohol; OAI SSI, overall incidence of SSIs; RCT, randomized controlled trial; SSI, surgical site infection

**a** SSI definitions: CDC, pathogens replicating within the wound cause infection at the surgical site, resulting in tissue damage; B, purulent discharge from the incision site, wound dehiscence, localized pain or tenderness, localized swelling, and erythema or heat within 30 days following caesarean section; C, for example, purulent / serous discharge from the wound, redness of the surrounding area, pain associated with discharge, increased local temperature within 10 days of the surgery; D, need for antibiotics or surgical intervention within 6 weeks of the surgery; E, presence of purulent drainage, cellulitis, or the need for incision and drainage, or treatment with antibiotics for a clinical diagnosis of infection, within 2 weeks of the surgery; F, wound healing disorders: when CDC criteria were met; SSI are diagnosed when CDC criteria plus 1 of the following criteria are met: 1) need for antibiotic therapy, 2) need for surgical intervention, 3) positive microbiological culture of swabs taken intraoperatively; G, patient reporting a need to use an antibiotic for a wound infection or documented wound infection in the medical record at the outpatient visit within 30 days of discharge; H, purulent discharge from the surgical wound or if a surgeon judges it to be infected and opens it (incisional), within 1 month of the surgery

### Quality assessment of the included studies

**FIGURE 2:**
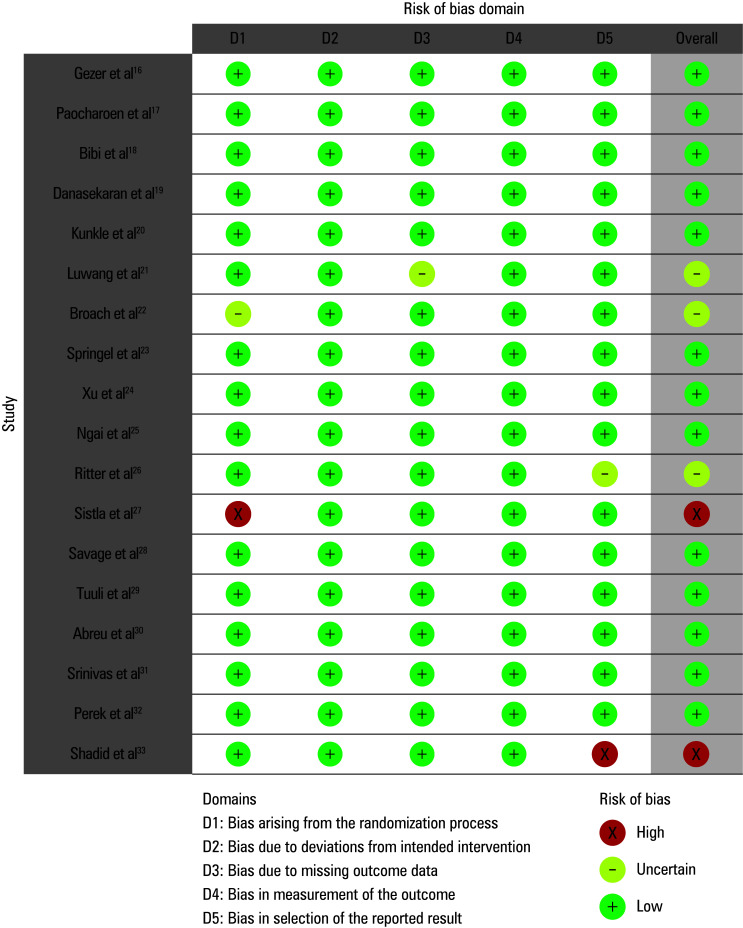
Risk of bias summary plot

**FIGURE 3:**
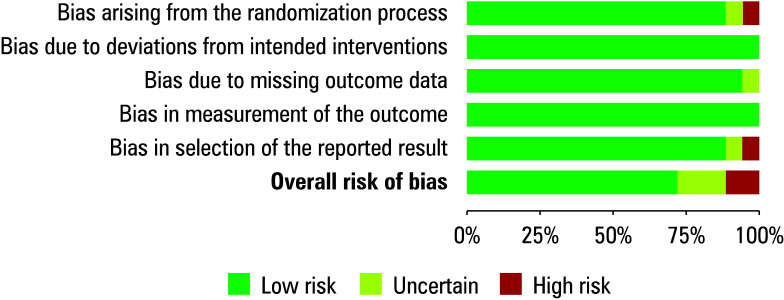
Traffic light plot for risk of bias assessment

A risk of bias was evaluated with a predetermined questionnaire to ascertain the study’s overall quality score. TABLE 3  displays the results of the risk of bias assessment for each of 18 included RCTs. Our meta‑analysis exhibits a minimal risk of bias, as indicated by the traffic light plot (FIGURE 2) and the summary plot for bias assessment (FIGURE 3). The risk of bias was low for 13 RCTs and moderate for 3.^21^^;^^22^^;^^26^ This is attributed to issues with missing outcome data, randomization method, and the selection of reported outcomes. The other 2 RCTs^27^^;^^33^ demonstrated a high risk of bias pertaining to the randomization method and bias in the selection of reported outcomes, respectively.

**TABLE 3:** Risk assessment of the included studies

Study	Gezer et al^16^	Paocharoen et al^17^	Bibi et al^18^	Danasekaran et al^19^	Kunkle et al^20^	Luwang et al^21^	Broach et al^22^	Springel et al^23^	Xu et al^24^	Ngai et al^25^	Ritter et al^26^	Sistla et al^27^	Savage et al^28^	Tuuli et al^29^	Abreu et al^30^	Srinivas et al^31^	Perek et al^32^	Shadid et al^33^
Was a consecutive or random sample of patients enrolled?	Y	Y	Y	Y	Y	Y	Y	Y	Y	Y	Y	Y	Y	Y	Y	Y	Y	Y
Did the study avoid inappropriate exclusions	Y	Y	Y	Y	Y	Y	Y	Y	Y	Y	Y	Y	Y	Y	Y	Y	Y	Y
Did all patients receive the same reference standard?	Y	Y	Y	Y	Y	Y	Y	Y	Y	Y	Y	Y	Y	Y	Y	Y	Y	Y
Were all patients included in the analysis?	N	N	N	N	N	N	N	N	N	N	N	N	N	N	N	N	N	N
Was the sample frame appropriate to address the target population?	Y	Y	Y	Y	Y	Y	Y	Y	Y	Y	Y	Y	Y	Y	Y	Y	Y	Y
Were the study participants sampled in an appropriate way?	Y	Y	Y	Y	Y	Y	Y	Y	Y	Y	Y	Y	Y	Y	Y	Y	Y	Y
Were the study participants and the setting described in detail?	Y	Y	Y	Y	Y	Y	Y	Y	Y	Y	Y	Y	Y	Y	Y	Y	Y	Y
Were valid methods used for identification of the condition?	Y	Y	Y	Y	Y	Y	Y	Y	Y	Y	Y	Y	Y	Y	Y	Y	Y	Y
Was the condition measured in a standard, reliable way for all participants?	Y	Y	Y	Y	Y	Y	Y	Y	Y	Y	Y	Y	Y	Y	Y	Y	Y	Y

Abbreviations: N, no; Y, yes

### Findings derived from the statistical investigation

In all, 10587 surgical patients from 18 RCTs were included in the current meta‑analysis to evaluate the efficacy of different concentrations of preoperative antiseptic (0.5%, 2%–2.5%, and 4% CHA) vs aqueous / alcoholic iodine for prevention of postoperative SSIs. The conclusions on the primary study outcomes are listed below.

### Effect on overall incidence of postoperative surgical site infections

**FIGURE 4:**
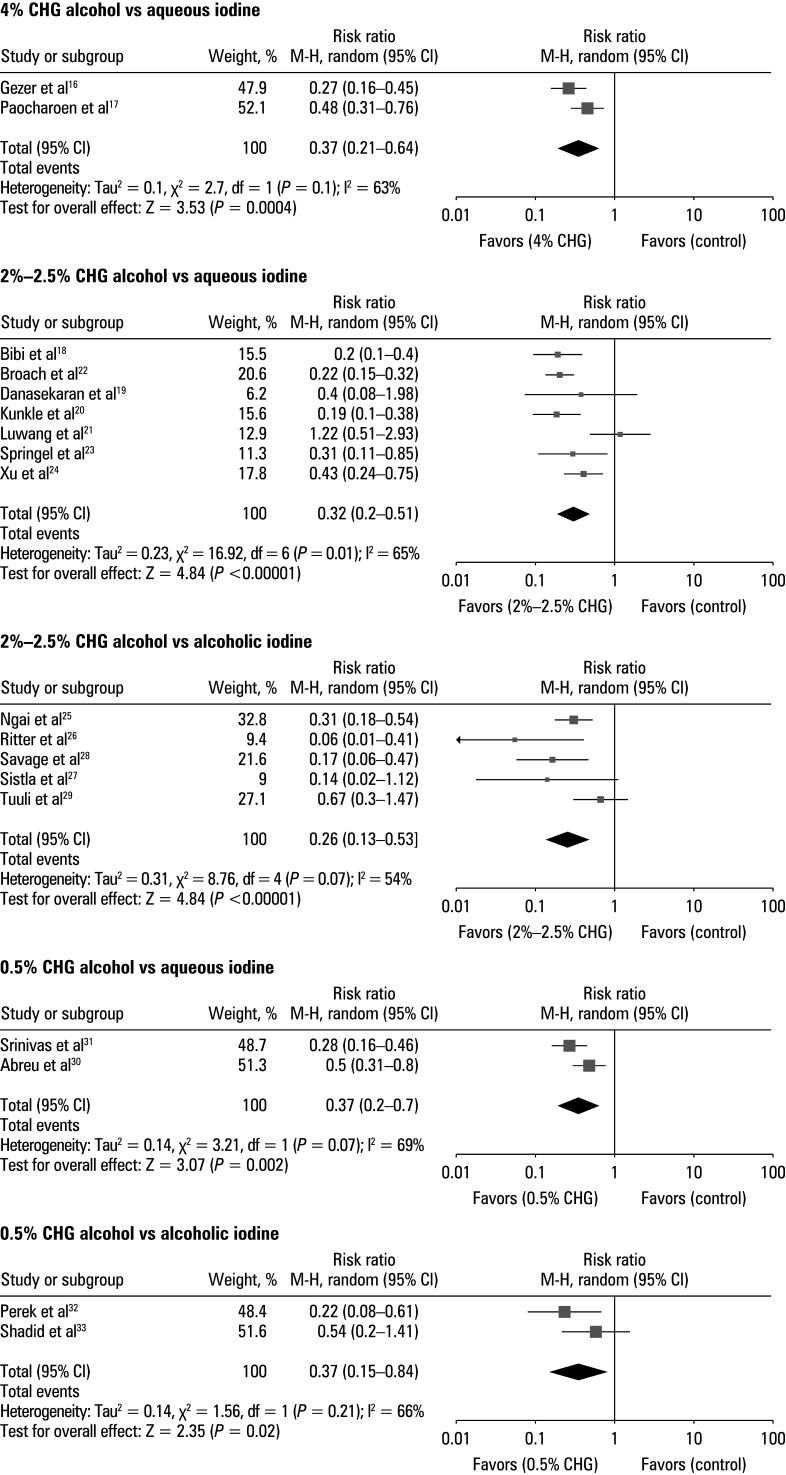
Forest plot for overall incidence of surgical site infections for different concentrations of chlorhexidine alcohol vs aqueous or alcoholic iodine

Abbreviations: df, degree of freedom; M-H, Mantel– Haenszel fixed-effects model; others, see TABLE 2

**FIGURE 5:**
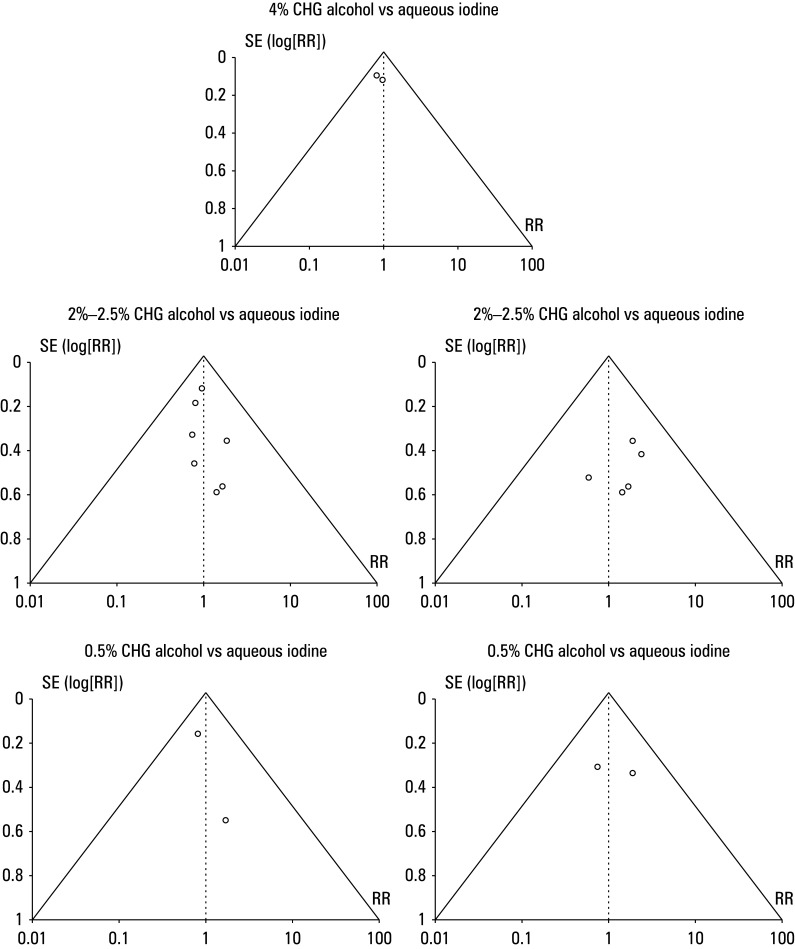
Funnel plot for overall incidence of surgical site infections for different concentrations of chlorhexidine alcohol vs aqueous or alcoholic iodine

Abbreviations: RR, risk ratio; SE, standard error; others, see TABLE 2

The risk ratio (RR) of SSI incidence in the CHA and iodine group (IG) was calculated using event data from the included studies FIGURE 4. The patients treated with CHA demonstrated a lower incidence of postoperative SSIs than those treated with iodine. For a comparison between 4% CHA and aqueous iodine, the RR was 0.37 (95% CI, 0.21–0.64; tau^2^ = 20.1; χ^2^ = 2.7; degree of freedom [df] = 1; Z = 3.53; I^2^ = 63%; *P* <0.01). In comparison with aqueous iodine, the RR for the 2%–2.5% CHA group was 0.32 (95% CI, 0.2–0.51; tau^2^ = 20.23; χ^2^ = 16.92; df = 6; Z = 4.84; I^2^ = 65%; *P* <0.01). For 2%–2.5% CHA vs alcoholic iodine, the RR was 0.26 (95% CI, 0.13–0.53; tau^2^ = 20.31; χ^2^ = 8.76; df = 4; Z = 3.73; I^2^ = 54%; *P* <0.01). The RR was 0.37 (95% CI, 0.2–0.7; tau^2^ = 20.14; χ^2^ = 3.21; df = 1; Z = 3.07; I^2^ = 69%; *P* = 0.002) for 0.5% CHA vs aqueous iodine and 0.35 (95% CI, 0.15–0.84; tau^2^ = 20.14; χ^2^ = 1.56; df = 1; Z = 2.35; I^2^ = 66%; *P* = 0.02) for 0.5% CHA vs alcoholic iodine. Furthermore, a symmetrical shape of all funnel plots in FIGURE 5 and insignificant results of the Egger test (*P* = 0.141 for 4% CHA, *P* = 0.214 for 2%–2.5% CHA vs aqueous iodine, *P* = 0.225 for 2%–2.5% CHA vs alcoholic iodine, *P* = 0.158 for 0.5% CHA vs aqueous iodine, and *P* = 0.301 for 0.5% CHA vs alcoholic iodine), indicated a low risk of publication bias.

**FIGURE 6:**
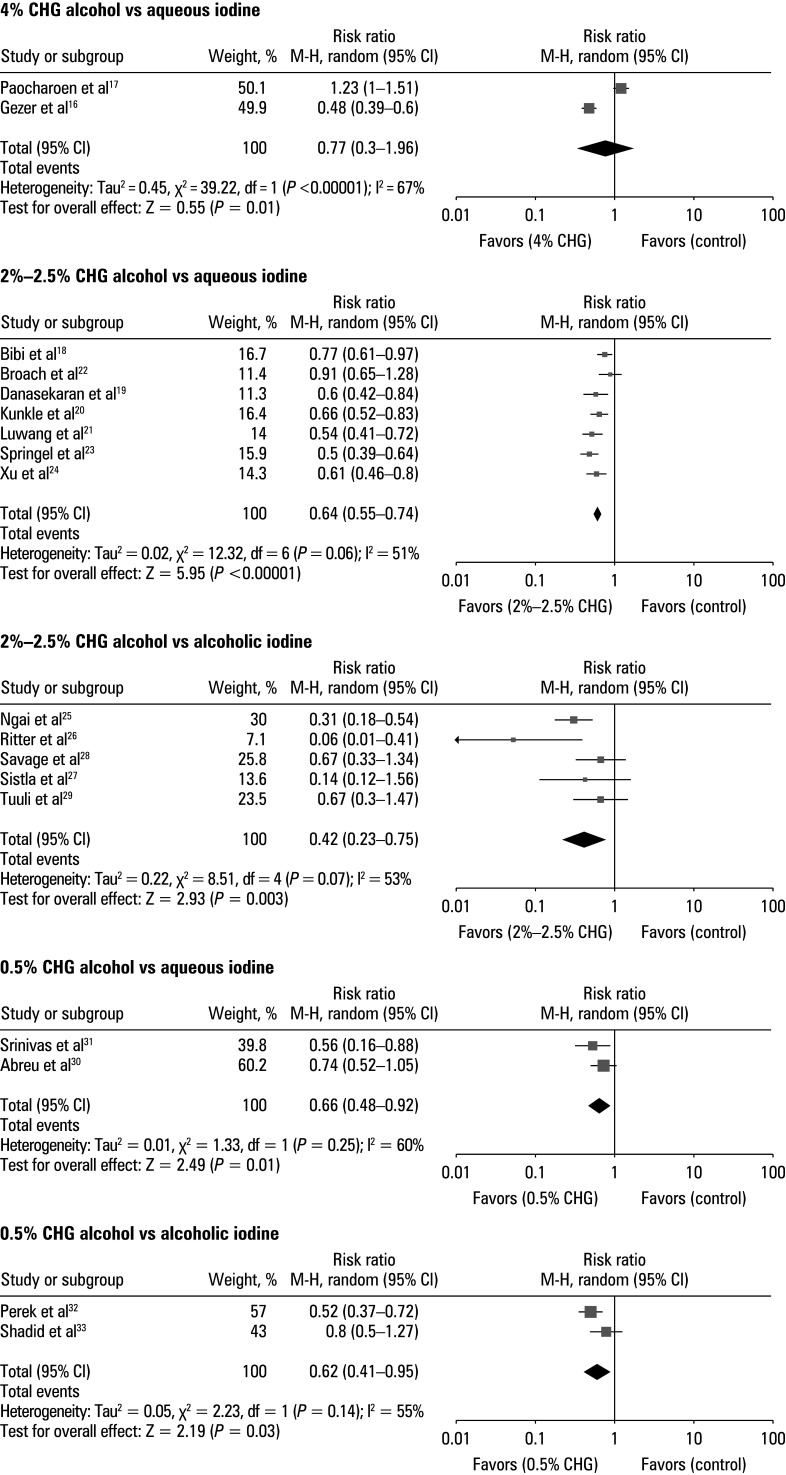
Forest plot for antimicrobial effect of different concentrations of chlorhexidine alcohol vs aqueous or alcoholic iodine

Abbreviations: see FIGURE 4

**FIGURE 7:**
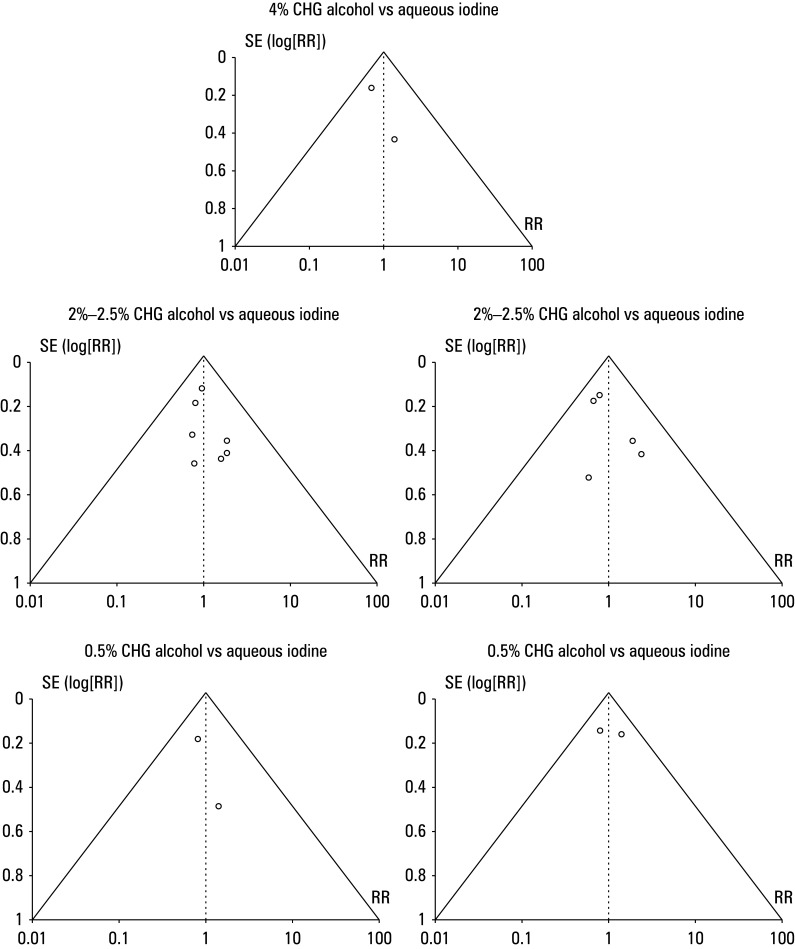
Funnel plot for antimicrobial effect of different concentrations of chlorhexidine alcohol vs aqueous or alcoholic iodine

Abbreviations: see TABLE 2  and FIGURE 5

Antimicrobial effect of different concentrations of chlorhexidine alcohol To assess the effect of different concentrations of CHA and aqueous or alcoholic iodine on antimicrobial activity, event data extracted from the included studies were used to calculate RR (FIGURE 6). The patients treated with CHA were shown to have a reduced growth of microorganisms on a swab taken from the surgical sites, as compared with those treated with iodine. For 4% CHA vs aqueous iodine, the RR was 0.77 (95% CI, 0.3–1.96; tau^2^ = 20.45; χ^2^ = 39.22; df = 1; Z = 0.55; I^2^ = 67%; *P* = 0.01). For CHA 2%–2.5% vs aqueous iodine, the RR was 0.64 (95% CI, 0.55–0.74; tau^2^ = 20.02; χ^2^ = 12.32; df = 6; Z = 5.95; I^2^ = 51%; *P* <0.01), while for CHA 2%–2.5% and alcoholic iodine it was 0.42 (95% CI, 0.23–0.75; tau^2^ = 20.22; χ^2^ = 8.51; df = 4; Z = 2.93; I^2^ = 53%; *P* = 0.003). For 0.5% CHA vs aqueous iodine the RR was 0.66 (95% CI, 0.48–0.92; tau^2^ = 20.01; χ^2^ = 1.33; df = 1; Z = 2.49; I^2^ = 60%; *P* = 0.01), and for 0.5% CHA vs alcoholic iodine it was 0.62 (95% CI, 0.41–0.95; tau^2^ = 20.05; χ^2^ = 2.23; df = 1; Z = 2.19; I^2^ = 55%; *P* = 0.03). Moreover, a symmetrical shape of all associated funnel plots in FIGURE 7 and insignificant results of the Egger test (*P* = 0.13 for 4% CHA; *P* = 0.37 for 2%–2.5% CHA vs aqueous iodine; *P* = 0.12 for 2%–2.5% CHA vs alcoholic iodine; *P* = 0.26 for 0.5% CHA vs aqueous iodine; and *P* = 0.37 for 0.5% CHA vs alcoholic iodine) confirmed a low risk of the publication bias.

### Adverse effects associated with the use of different preoperative antiseptics on the surgical sites

**FIGURE 8:**
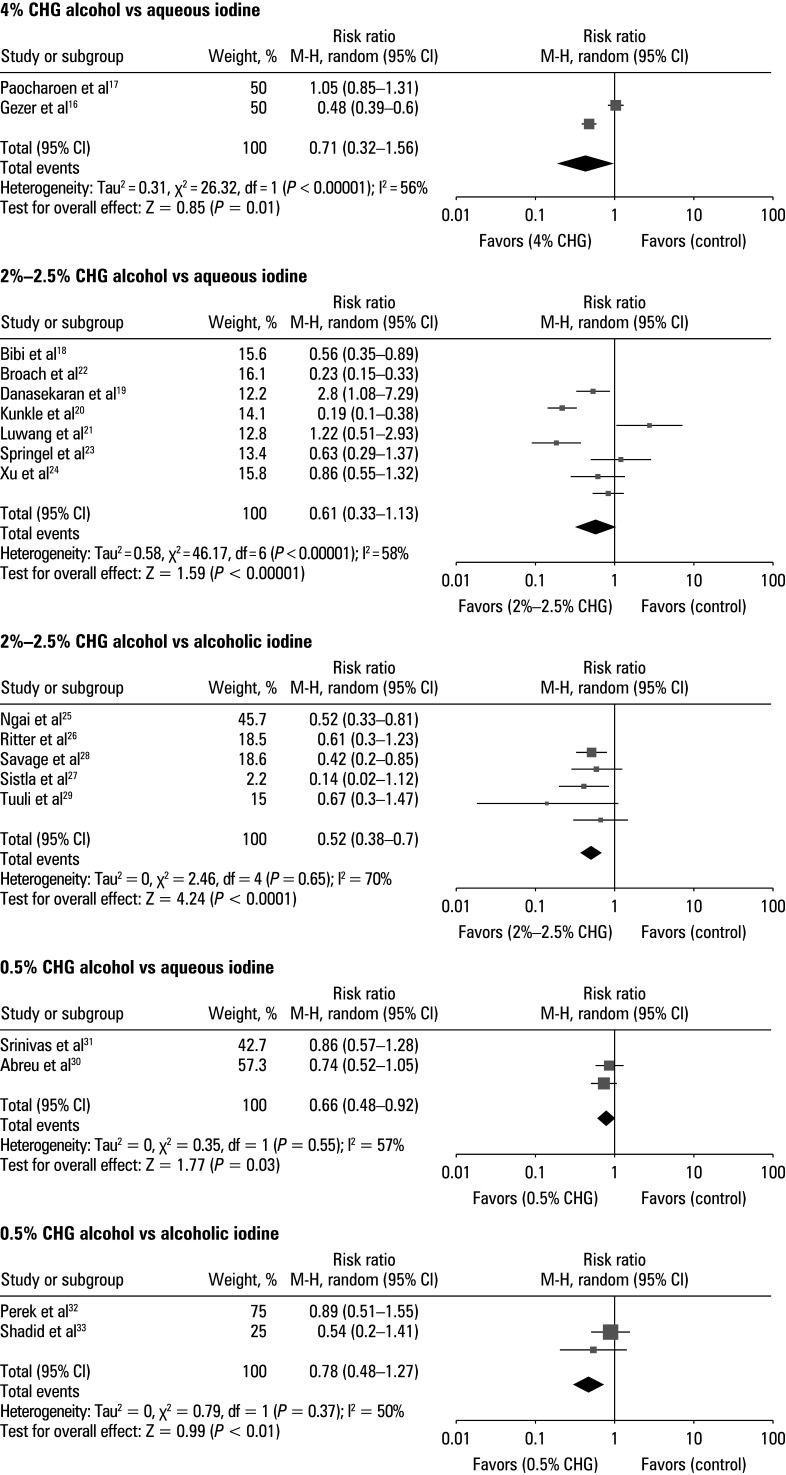
Forest plot for adverse events associated with different concentrations of chlorhexidine alcohol vs aqueous or alcoholic iodine

Abbreviations: see FIGURE 4

**FIGURE 9:**
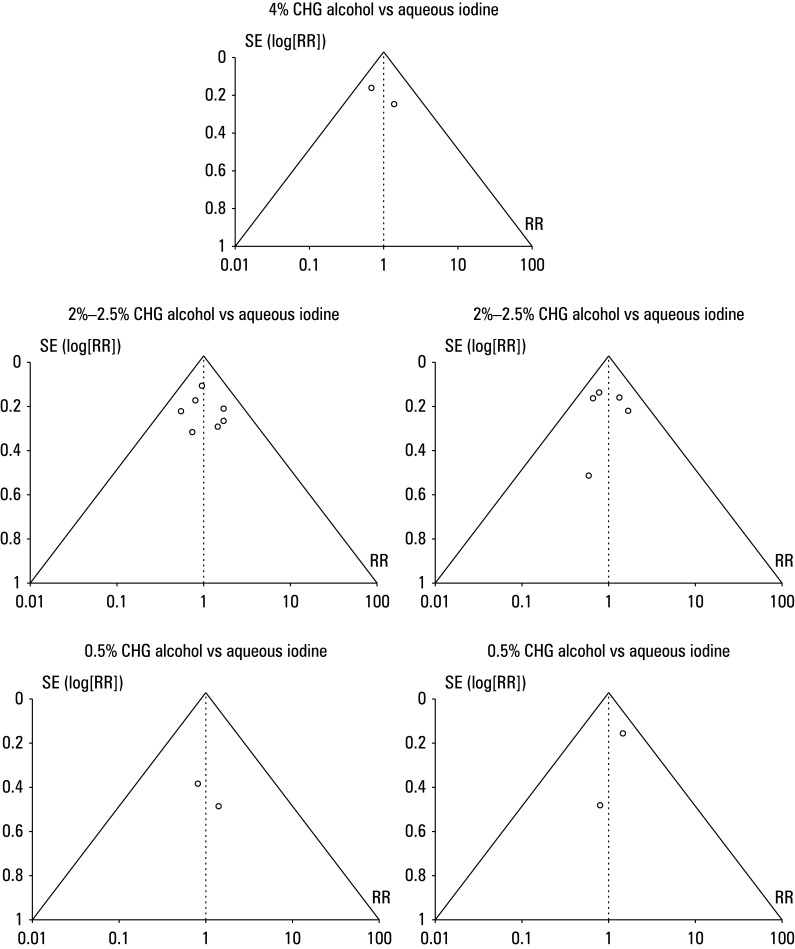
Funnel plot for adverse events associated with different concentrations of chlorhexidine alcohol vs aqueous or alcoholic iodine

Abbreviations: see FIGURE 5

To evaluate the adverse events associated with the use of various concentrations of CHA and either aqueous or alcoholic iodine, the data extracted data from the included studies were utilized to calculate the RR for the patients treated with CHA and iodine (FIGURE 8). The CHA‑treated patients exhibited lower incidence of adverse effects, such as skin irritation, erythema, allergic reactions, and pain, as compared with the iodine‑treated individuals. RR for 4% CHA vs aqueous iodine was 0.71 (95% CI, 0.32–1.56; tau^2^ = 20.31; χ^2^ = 26.32; df = 1; Z = 0.85; I^2^ = 56%; *P* = 0.01). RR for aqueous iodine vs 2%–2.5% CHA was 0.61 (95% CI, 0.33–1.13; tau^2^ = 20.58; χ^2^ = 46.17; df = 6; Z = 1.59; I^2^ = 58%; *P* = 0.02), and for 2%–2.5% CHA and alcoholic iodine it was 0.52 (95% CI, 0.38–0.7; tau^2^ = 20; χ^2^ = 2.46; df = 4; Z = 4.24; I^2^ = 70%; *P* <0.01). For 0.5% CHA, RR was 0.79 (95% CI, 0.61–1.03; tau2 = 20; χ^2^ = 0.35; df = 1; Z = 1.77; I^2^ = 57%; *P* = 0.03) for aqueous iodine and 0.78 (95% CI, 0.48–1.27; tau^2^ = 20; χ^2^ = 0.79; df = 1; Z = 0.99; I^2^ = 50%; *P* <0.01) for alcoholic iodine. In addition, the symmetrical shape of all associated funnel plots in FIGURE 9, along with insignificant results of the Egger test (*P* = 0.31 for 4% CHA, *P* = 0.24 for 2%–2.5% CHA vs aqueous iodine, *P* = 0.28 for 2%–2.5% CHA vs alcoholic iodine, *P* = 0.13 for 0.5% CHA vs aqueous iodine, and *P* = 0.22 for 0.5% CHA vs alcoholic iodine), indicated a low risk of the publication bias.

### Hierarchical summary receiver operating characteristic curve plot for test accuracy of the included studies

**FIGURE 10:**
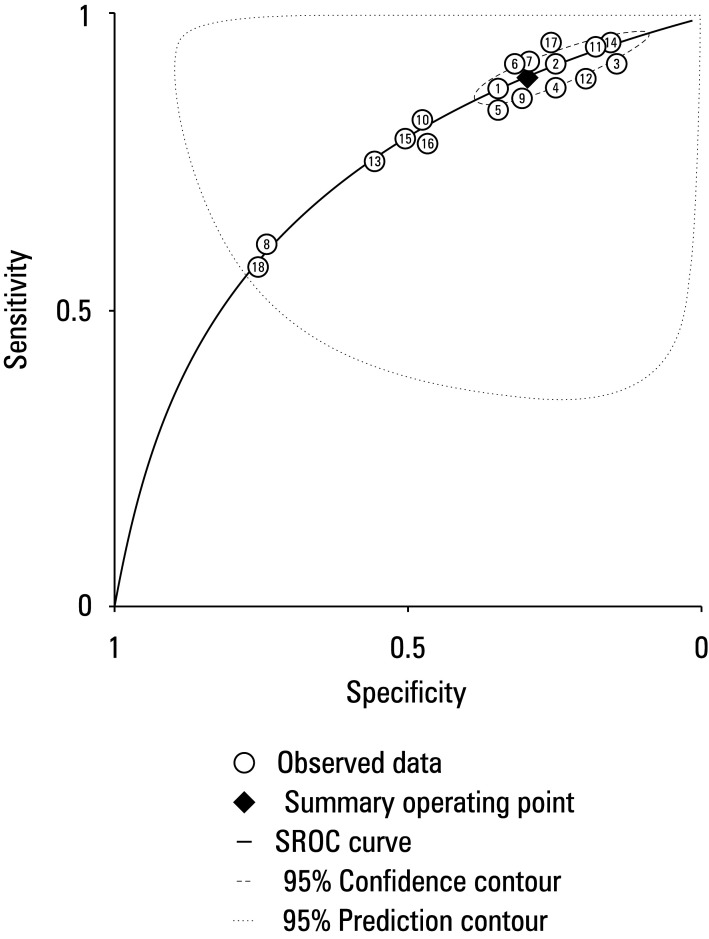
Hierarchical summary receiver operating characteristic curve (SROC) plot

We assessed the subjective accuracy of all included studies using the HSROC plot, as illustrated in FIGURE 10. The curve is presented as a straight line, with each research area represented by a circular shape. The square represents the point estimate that corresponds with the summary sensitivity and specificity, while the dashed line indicates the associated 95% CI. The regression line represents the curve that summarizes the overall diagnostic accuracy. The included studies demonstrated a high level of test accuracy as inferred by the clustering of all the data points in the upper left corner, wherein the sensitivity values are near 1 and the specificity values are near 0. The area under the curve of the HSROC was 0.92, with 95% CI of 0.81–0.97. This indicates the inherent reliability of our assessment.

## DISCUSSION

The rationale for hospital surgeries may differ. They may involve removal or repair of diseased tissues or organs, relieving an obstruction, reposition of structures to their normal position, and redirection of blood vessels via bypass surgery. Additionally, they may be aimed at alleviating or preventing pain, diagnosing a problem, or improving a body function.^50^^;^^51^ Laparoscopic surgery, which was one of the first types of minimally‑invasive surgery, is preferred by health care providers for a wider range of everyday operations due to its improved surgical outcomes and lower cost. The procedure entails performing small incisions and insertion of a tube equipped with a camera and a light source (laparoscope). It allows for producing smaller scars, accelerates discharge from a hospital, reduces pain during healing, shortens recovery, and limits the risk of SSIs and hemorrhage.^52^^;^^53^

Numerous studies have indicated that minimally‑invasive laparoscopic surgery reduces the prevalence of SSIs as a consequence of faster healing and small incisions.^54^^;^^55^^;^^56^ However, this decrease does not entirely prevent SSIs. Consequently, it is imperative to cleanse the skin to minimize the risk of SSIs by removing as many microorganisms as possible. The skin cleansers or skin antiseptics help prevent SSIs by removing debris from the skin and cleansing it, reducing the number of resident and transient microbes to an irreducible minimum, and preventing the growth of microbes that could potentially enter the cuts incurred during a surgical procedure.^57^^;^^58^

The use of preoperative skin antiseptics is a widely accepted and prevalent practice demonstrated as beneficial in the prevention of SSIs.^59^ Nevertheless, there is an ongoing debate regarding the most effective antiseptic that can be used to prevent SSIs. Various guidelines recommend CHA as a prospective surgical skin preparation to prevent SSIs. Chlorhexidine efficacy is attributed to its dual mode of action, which encompasses both bacteriostatic and bactericidal activity. The efficacy of chlorhexidine in removing bacterial cells depends on its concentration, as it disrupts the bacterial cell membranes. Chlorhexidine can virtually eradicate all gram‑positive and gram‑negative microorganisms within 30 seconds of in vitro treatment.^60^^;^^61^^;^^62^

Therefore, this systematic review and meta‑analysis examined the efficacy of different preoperative skin antiseptic solutions and their concentrations on the incidence of SSIs in adult patients undergoing any type of surgical procedures. We evaluated the efficacy of 2 types solutions: CHA and aqueous or alcoholic iodine. Additionally, we investigated the impact of different concentrations of CHA on the rate of SSIs. We evaluated the efficacy of aqueous or alcoholic iodine solutions in preventing postoperative SSIs in comparison with 0.5%, 2%–2.5%, and 4% CHA. While 0.5% and 4% CHA was efficient, the most effective CHA concentration was 2%– 2.5%, as confirmed by lower RR, significant *P* values, and 95% CIs. In comparison to aqueous iodine, the effects of aqueous chlorhexidine and alcoholic iodine were comparable. Furthermore, 2%–2.5% CHA was determined as equally efficacious in both clean and nonclean surgical procedures, as it provides high and broad‑spectrum antimicrobial activity and durable effects.

Below we summarize the main conclusions from the included studies.

In a cohort of 220 patients undergoing surgery for malignant or premalignant conditions, Gezer et al^16^ compared 10% PI at 25 °C, 10% PI at 37 °C (warm PI), 4% chlorhexidine gluconate with alcohol at 25 °C (CH), and 4% chlorhexidine gluconate with alcohol at 37 °C (warm CHA). The antiseptic effect was not demonstrated for chlorhexidine solutions, but the authors reported that SSI rates can be reduced by reheating PI. In malignant and premalignant gynecologic operations, no significant difference in SSI prevention efficacy was observed when both groups of PI were compared to both groups of CHA. Paocharoen et al^17^ investigated 500 patients randomly assigned to PI and chlorhexidine treatment. Colonization by bacteria and postoperative surgical wound infections were substantially diminished in the chlorhexidine group.

Bibi et al^18^ reported on 2 groups of patients from different hospitals (n = 220 and n = 168), in which SSIs were identified in 22 individuals (10%) from the first group treated with PI and 12 patients (7.1%) from the second group treated with chlorhexidine gluconate in alcohol (*P* = 0.32). The primary bacterium associated with SSIs was *Pseudomonas aeruginosa* (23.5%), followed by *Staphylococcus aureus* (17.6%). Similarly, Danasekaran et al^19^ reported that a S. *aureus* infection was initially present in 1.67% of the chlorhexidine‑alcohol group group (CAG) and in 10% of the iodine group. Bacterial colonization was reduced in both groups following application of the antiseptic agents, with the CAG group exhibiting greater decrease. In their study of 60 participants, with 55% belonging to the PI group, Kunkle et al^20^ discovered that women in the PI group were 7 times more likely to have a positive culture than women in the CAG group (16/33; 48.5% vs 3/27; 11.1%), with odds ratio (OR) of 7.53 (95% CI, 1.67–38.83; *P* = 0.002). Additionally, they reported that the prevalence of positive bacterial cultures obtained at the site of the skin incision was higher in the PI group. Luwag et al^21^ reported *Escherichia coli*, *Klebsiella pneumoniae*, and *Acinetobacter baumannii*, as the microorganisms most frequently isolated from a SSI. The rate of SSI in the CHA group was below 5.4%, while in the PI group it was 8.6%.

In a cohort of 788 patients, Broach et al^22^ found that the overall SSI rate varied between iodine in isopropenyl acetate (IPA; 18.7%) and CHA (15.9%). Additionally, they determined that IPA did not meet the criteria for overall SSI prevention in clean contaminated surgery when compared with CHA. In a study of 932 participants (461 assigned to CHA and 471 to PI disinfection), Springel et al^23^ reported a SSI rate of 6.3% in the CHA group and 7% in the PI group. Simultaneously, Xu et al^24^ discovered that in a cohort of 159 patients aged above 18 years, the overall risk of SSI in the 2%–2.5% CHA group (3.8%) was lower than in the aqueous iodine group (23%). Ngai et al^25^ reported that the overall SSI rate was lower in the CHA group (4.5%) than in the PI group in their trial involving 1404 women (n = 463 in the PI group, n = 474 in the CHA group, or n = 467 in both groups). Ritter et al^26^ reported that the SSI rate was considerably higher in the povidone‑iodine group (12.6%) than in the CAG group (4.5%) (*P* = 0.02).

Sistla et al^27^ found that the infection rates in the PI and CHA groups were not substantially different (9.5% vs 7%). Additionally, both antiseptics significantly reduced the skin bacterial colony counts. Comparing 2%–2.5% CHA with alcoholic iodine Savage et al^28^ discovered that chlorhexidine was equally effective in eradication of common bacterial infections in 100 participants aged over 18 years. In their study of 1147 patients, Tuuli et al^29^ discovered that SSIs were diagnosed in 23 patients (4%) in CAG and in 42 individuals (7.3%) in the IG group (RR, 0.55; 95% CI, 0.34–0.9; *P* = 0.02). They also showed that using CHA as preoperative skin antiseptic significantly decreased SSI probability.

Abreau et al^30^ conducted an RCT that included 70 patients who underwent open surgery for benign prostatic hyperplasia. The authors reported that skin antisepsis with CHA was more effective than that with 0.5% PI. Srinivas et al^31^ randomly assigned 351 patients aged 18–70 years to chlorhexidine or PI skin preparation group. They found that the incidence of SSIs following clean‑contaminated upper‑abdominal surgeries was lower with chlorhexidine than with PI. Perek et al^32^ conducted a randomized trial that involved 91 consecutive patients, at a mean (SD) age of 66.2 (9.9) years, undergoing elective cardiac surgery. The study found that chlorhexidine in 70% ethanol was a more effective surgical site antiseptic than PI in 50% propanol. Shadid et al^33^ compared the efficacy of 5% chlorhexidine in 70% alcohol and 1% iodine in 70% alcohol in reducing positive cultures prior to elective foot surgery. The study also aimed to investigate any wound complications, infections, and allergic reactions. It found that the quantity of positive cultures in elective foot surgery was reduced by both antiseptics.

Previous systematic evaluations and network meta‑analyses also identified 2%–2.5% CHA as the most effective antiseptic for reducing the rate of SSIs. Wade et al^63^ conducted a comprehensive study and network meta‑analysis indicating that 4% CHG is the most effective antiseptic for minimizing SSIs. Nonetheless, this network meta‑analysis was confined to sterile procedures and inaccurately classified the included studies based on the CHG 4%–5%. In their systematic review and meta‑analysis, Hasegawa et al^64^ clarified the potential advantages of an alcoholic solution of chlorhexidine at a concentration of 0.5% or higher for surgical skin preparation in order to prevent SSI. Similarly, Jalalzadeh et al^65^ performed a systematic review, grade assessment, and network meta‑analysis and showed that the most effective method of preventing SSIs in adult patients undergoing surgery is to prepare the epidermis with 2% to 5% CHA, irrespective of the type of the wound. Our meta‑analysis, which yielded significant results and RR of postoperative incidence of SSIs and adverse events below 1, indicated that the use of 2%–2.5% CHA is substantially more effective in reducing SSIs when compared with 4% and 0.5% CHA or either aqueous or alcoholic iodine in any surgical procedure.

### Limitations

This study’s strength lies in the implementation of precise search criteria, including the examination of “postoperative surgical site infections,” “preoperative skin antiseptics,” and “incidence of surgical site infections” across various databases. However, it is necessary to address specific limitations. First, our study may have had a selection bias due to exclusion of many studies. Furthermore, this meta‑analysis includes only 18 publications, which demonstrate significant heterogeneity. In addition, we did not take into account the risk factors for SSIs in different surgeries, such as age, obesity, concurrent disorders (eg, diabetes or hypertension), and individuals with impaired immune systems. Also, each subgroup had a restricted number of participants. Our study only focused on the effect of skin preparation as a preventive approach for SSIs, not considering timing of the surgical antimicrobial prophylaxis and irrigation of the wound site. Hence, it is critical to conduct additional research using a larger sample size that takes into account these risk factors to determine the advantages of using 2%–2.5% CHA vs 0.5% and 4% CHA, as well as aqueous and alcoholic iodine solutions for skin disinfection and SSI prevention.

## CONCLUSIONS

The evidence presented in this meta‑analysis supports the efficacy of all concentrations of CHA antiseptics in preventing SSIs in adult patients undergoing surgery, as compared with conventional aqueous or alcoholic iodine. However, we found that the number of SSIs could be reduced more effectively by utilizing 2%–2.5% CHA instead of 4% or 0.5% CHA. The former concentration exhibited a more potent antimicrobial effect and induced fewer adverse effects. We did not observe any disparities in the efficacy of CHA for clean and nonclean surgery. Thus, we can infer that preoperative skin cleansing with chlorhexidine effectively reduces the risk of postoperative SSIs and bacterial colonization in any type of surgery. Nevertheless, the study is restricted by the inclusion of a relatively small number of RCTs. Therefore, future research ought to include a greater number of RCTs and larger sample sizes in order to validate these findings and strengthen the overall evidence.

## References

[BIBR-1] F Mateo Vallejo (2012). General surgery: present and future. Int J Surg.

[BIBR-2] John A, I Caturegli, NS Kubicki, SM Kavic (2020). The rise of minimally invasive surgery: 16 year analysis of the progressive replacement of open surgery with laparoscopy. JSLS.

[BIBR-3] Spagnolo A.M., Ottria G., Amicizia D. (2013). Operating theatre quality and prevention of surgical site infections. J Prev Med Hyg.

[BIBR-4] Mohan N., Gnanasekar D., Tk S., Ignatious A. (2023). Prevalence and risk factors of surgical site infections in a teaching medical college in the trichy district of India. Cureus.

[BIBR-5] Mekhla Borle F.R. (2019). Determinants of superficial surgical site infections in abdominal surgeries at a rural teaching hospital in central India: a prospective study. J Family Med Prim Care.

[BIBR-6] Bucataru A., Balasoiu M., Ghenea A.E. (2024). Factors contributing to surgical site infections: a comprehensive systematic review of etiology and risk factors. Clinics and Practice.

[BIBR-7] Alfonso‐Sanchez J.L., Martinez I.M., Martín‐Moreno J.M. (2017). Analyzing the risk factors influencing surgical site infections: the site of environmental factors. Can J Surg.

[BIBR-8] (2018). Global guidelines for the prevention of surgical site infection.

[BIBR-9] (2019). National Institute for Health and Care Excellence. Surgical site infections: prevention and treatment. https://www.nice.org.Uk/guidance/.

[BIBR-10] Berríos‐Torres S.I., Umscheid C.A., Bratzler D.W. (2017). Centers for Disease Control and prevention guideline for the prevention of surgical site infection, 2017. JAMA Surg.

[BIBR-11] Charehbili A., Koek M.B.G., Otterloo J.C.A. (2019). Cluster‐randomized crossover trial of chlorhexidine‐alcohol versus iodine‐alcohol for prevention of surgical‐site infection (SKINFECT trial. BJS Open.

[BIBR-12] Peel T.N., Watson E., Lee S.J. (2021). Randomised controlled trials of alcohol‐based surgical site skin preparation for the prevention of surgical site infections: systematic review and meta‐analysis. J Clin Med.

[BIBR-13] Mastrocola M., Matziolis G., Böhle S. (2021). Meta‐analysis of the efficacy of preoperative skin preparation with alcoholic chlorhexidine compared to povidone iodine in orthopedic surgery. Sci Rep.

[BIBR-14] Lee I., Agarwal R.K., Lee B.Y. (2010). Systematic review and cost analysis comparing use of chlorhexidine with use of iodine for preoperative skin antisepsis to prevent surgical site infection. Infect Control Hosp Epidemiol.

[BIBR-15] Majidipour N., Abdeyazdan Z., Zargham‐Boroujeni A. (2013). Chlorhexidine or povidone‐iodine: which solution is more effective on skin colonization in neonates?. Iran J Nurs Midwifery Res.

[BIBR-16] Gezer S., Yalvaç H.M., Güngör K., Yücesoy İ. (2020). Povidone‐iodine vs chlorhexidine alcohol for skin preparation in malignant and premalignant gynaecologic diseases: a randomized controlled study. Eur J Obstet Gynecol Reprod Biol.

[BIBR-17] Paocharoen V., Mingmalairak C., Apisarnthanarak A. (2009). Comparison of surgical wound infection after preoperative skin preparation with 4% chlorhexidine [correction of chlohexidine] and povidone iodine: a prospective randomized trial. J Med Assoc Thai.

[BIBR-18] Bibi S., Shah S.A., Qureshi S. (2015). Is chlorhexidine‐gluconate superior than povidone‐iodine in preventing surgical site infections? A multicenter study. J Pak Med Assoc.

[BIBR-19] Danasekaran G., Rasu S., Palani M. (2017). A study of comparative evaluation of preoperative skin preparation with chlorhexidine alcohol versus povidone iodine in prevention of surgical site infections. J Evid Based Med Healthc.

[BIBR-20] Kunkle C.M., Marchan J., Safadi S. (2015). Chlorhexidine gluconate versus povidone iodine at cesarean delivery: a randomized controlled trial. J Matern Fetal Neonatal Med.

[BIBR-21] Luwang A.L., Saha P.K., Rohilla M. (2021). Chlorhexidine‐alcohol versus povidone‐iodine as preoperative skin antisepsis for prevention of surgical site infection in cesarean delivery‐a pilot randomized control trial. Trials.

[BIBR-22] Broach R.B., Paulson E.C., Scott C., Mahmoud N.N. (2017). Randomized controlled trial of two alcohol‐based preparations for surgical site antisepsis in colorectal surgery. Ann Surg.

[BIBR-23] Springel E.H., Wang X.Y., Sarfoh V.M. (2017). A randomized open‐label controlled trial of chlorhexidine‐alcohol vs povidone‐iodine for cesarean antisepsis: the CAPICA trial. Am J Obstet Gynecol.

[BIBR-24] Xu P.Z., Fowler Goitz (2017). Prospective randomized trial comparing the efficacy of surgical preparation solutions in hand surgery. Hand.

[BIBR-25] Ngai I.M., Arsdale A., Govindappagari S. (2015). Skin preparation for prevention of surgical site infection after cesarean delivery: a randomized controlled trial. Obstet Gynecol.

[BIBR-26] Ritter B., Herlyn P.K.E., Mittlmeier T., Herlyn A. (2020). Preoperative skin antisepsis using chlorhexidine may reduce surgical wound infections in lower limb trauma surgery when compared to povidone‐iodine ‐ a prospective randomized trial. Am J Infect Control.

[BIBR-27] Sistla S.C., Prabhu G., Sistla S., Sadasivan J. (2010). Minimizing wound contamination in a “clean” surgery: comparison of chlorhexidine‐ethanol and povidone‐iodine. Chemotherapy.

[BIBR-28] Savage J.W., Weatherford B.M., Sugrue P.A. (2012). Efficacy of surgical preparation solutions in lumbar spine surgery. J Bone Joint Surg Am.

[BIBR-29] Tuuli M.G., Liu J., Stout M.J. (2016). A randomized trial comparing skin antiseptic agents at cesarean delivery. N Engl J Med.

[BIBR-30] Abreu D., Campos E., Seija V. (2014). Surgical site infection in surgery for benign prostatic hyperplasia: comparison of two skin antiseptics and risk factors. Surg Infect (Larchmt.

[BIBR-31] Srinivas A., Kaman L., Raj P. (2015). Comparison of the efficacy of chlorhexidine gluconate versus povidone iodine as preoperative skin preparation for the prevention of surgical site infections in clean‐contaminated upper abdominal surgeries. Surg Today.

[BIBR-32] Perek B., Lipski A., Stefaniak S., Jemielity M. (2013). QUALITY IN MEDICINE Comparative analysis of the antiseptic effectiveness of two commercially available skin disinfectants in cardiac surgery ‐ a preliminary report. Kardiochirurgia i Torakochirurgia Polska / Polish Journal of Thoracic and Cardiovascular Surgery.

[BIBR-33] Shadid M.B., Speth M.J.G.M., Voorn G.P., Wolterbeek N. (2019). Chlorhexidine 0.5%/70% alcohol and iodine 1%/70% alcohol both reduce bacterial load in clean foot surgery: a randomized, controlled trial. J Foot Ankle Surg.

[BIBR-34] Liberati A., Altman D.G., Tetzlaff J. (2009). The PRISMA statement for reporting systematic reviews and meta‐analyses of studies that evaluate healthcare interventions: explanation and elaboration. BMJ.

[BIBR-35] Brown D. (2020). A review of the PubMed PICO tool: using evidence‐based practice in health education. Health Promot Pract.

[BIBR-36] Higgins J.P., Altman D.G., Gøtzsche P.C. (2011). Cochrane Bias Methods Group; Cochrane Statistical Methods Group. The Cochrane Collaboration’s tool for assessing risk of bias in randomised trials. BMJ.

[BIBR-37] Sterne J.A., Egger M. (2001). Funnel plots for detecting bias in meta‐analysis: guidelines on choice of axis. J Clin Epidemiol.

[BIBR-38] Bowden J., Davey Smith G., Burgess S. (2015). Mendelian randomization with invalid instruments: effect estimation and bias detection through Egger regression. Int J Epidemiol.

[BIBR-39] Elovic A., Pourmand A. (2019). MDCalc medical calculator app review. J Digit Imaging.

[BIBR-40] Schmidt L., Shokraneh F., Steinhausen K., Adams C.E. (2019). Introducing RAPTOR: RevMan parsing tool for reviewers. Syst Rev.

[BIBR-41] O’Brien S.F., Yi Q.L. (2016). How do I interpret a confidence interval?. Transfusion.

[BIBR-42] George B.J., Aban I.B. (2016). An application of meta‐analysis based on DerSimonian and Laird method. J Nucl Cardiol.

[BIBR-43] Noma H., Misumi M., Tanaka S. (2023). Risk ratio and risk difference estimation in case‐cohort studies. J Epidemiol.

[BIBR-44] Dettori Norvell, DC Chapman (2021). Seeing the forest by looking at the trees: how to interpret a meta‐analysis forest plot. Global Spine J.

[BIBR-45] Huedo‐Medina T.B., Sánchez‐Meca J., Marín‐Martínez F., Botella J. (2006). Assessing heterogeneity in meta‐analysis: Q statistic or I2 index?. Psychol Methods.

[BIBR-46] Barili F., Parolari A., Kappetein P.A., Freemantle N. (2018). Statistical primer: heterogeneity, random‐ or fixed‐effects model analyses? Interact Cardiovasc Thorac Surg.

[BIBR-47] Kanters S. (2022). Fixed‐ and random‐effects models. Methods Mol Biol.

[BIBR-48] Andrade C. (2019). The P value and statistical significance: misunderstandings, explanations, challenges, and alternatives. Indian J Psychol Med.

[BIBR-49] Toft N., Nielsen S.S. (2009). Summary receiver operating characteristics (SROC) and hierarchical SROC models for analysis of diagnostic test evaluations of antibody ELISAs for paratuberculosis. Prev Vet Med.

[BIBR-50] Quene T.M., Bust L., Louw J. (2022). Global surgery is an essential component of global health. Surgeon.

[BIBR-51] Sacks G.D., Dawes A.J., Ettner S.L. (2016). Surgeon perception of risk and benefit in the decision to operate. Ann Surg.

[BIBR-52] Buia A., Stockhausen F., Hanisch E. (2015). Laparoscopic surgery: a qualified systematic review. World J Methodol.

[BIBR-53] Lee W.J., Chan C.P., Wang B.Y. (2013). Recent advances in laparoscopic surgery. Asian J Endosc Surg.

[BIBR-54] Kulkarni N., Arulampalam T. (2020). Laparoscopic surgery reduces the incidence of surgical site infections compared to the open approach for colorectal procedures: a meta‐analysis. Tech Coloproctol.

[BIBR-55] Utsumi M., Yamada T., Yamabe K. (2022). Differences in risk factors for surgical site infection between laparotomy and laparoscopy in gastrointestinal surgery. PLoS One.

[BIBR-56] Aimaq R., Akopian G., Kaufman H.S. (2011). Surgical site infection rates in laparoscopic versus open colorectal surgery. Am Surg.

[BIBR-57] Seidelman J.L., Mantyh C.R., Anderson D.J. (2023). Surgical site infection prevention: a review. JAMA.

[BIBR-58] Reichman D.E., Greenberg J.A. (2009). Reducing surgical site infections: a review. Rev Obstet Gynecol.

[BIBR-59] Poulin P., Chapman K., McGahan L. (2014). Preoperative skin antiseptics for preventing surgical site infections: what to do?. ORNAC J.

[BIBR-60] Lim K.S., Kam P.C. (2008). Chlorhexidine‐pharmacology and clinical applications. Anaesth Intensive Care.

[BIBR-61] Reichel M., Heisig P., Kohlmann T., Kampf G. (2009). Alcohols for skin antisepsis at clinically relevant skin sites. Antimicrob Agents Chemother.

[BIBR-62] Maiwald M., Chan E.S.Y. (2014). Pitfalls in evidence assessment: the case of chlorhexidine and alcohol in skin antisepsis. J Antimicrob Chemother.

[BIBR-63] Wade R.G., Burr N.E., McCauley G. (2021). The comparative efficacy of chlorhexidine gluconate and povidone‐iodine antiseptics for the prevention of infection in clean surgery: a systematic review and network meta‐analysis. Ann Surg.

[BIBR-64] Hasegawa T., Tashiro S., Mihara T. (2022). Efficacy of surgical skin preparation with chlorhexidine in alcohol according to the concentration required to prevent surgical site infection: meta‐analysis. BJS Open.

[BIBR-65] Jalalzadeh H., Groenen H., Buis D.R. (2022). Efficacy of different preoperative skin antiseptics on the incidence of surgical site infections: a systematic review, GRADE assessment, and network meta‐analysis. Lancet Microbe.

